# Genetic dissection of intraspecific variation in a male-specific sexual trait in *Drosophila melanogaster*

**DOI:** 10.1038/hdy.2016.63

**Published:** 2016-08-17

**Authors:** K M Cloud-Richardson, B R Smith, S J Macdonald

**Affiliations:** 1 Department of Molecular Biosciences, University of Kansas, Lawrence, KS, USA; 2 Center for Computational Biology, University of Kansas, Lawrence, KS, USA

## Abstract

An open question in evolutionary biology is the relationship between standing variation for a trait and the variation that leads to interspecific divergence. By identifying loci underlying phenotypic variation in intra- and interspecific crosses we can determine the extent to which polymorphism and divergence are controlled by the same genomic regions. Sexual traits provide abundant examples of morphological and behavioral diversity within and among species, and here we leverage variation in the *Drosophila* sex comb to address this question. The sex comb is an array of modified bristles or ‘teeth' present on the male forelegs of several Drosophilid species. Males use the comb to grasp females during copulation, and ablation experiments have shown that males lacking comb teeth typically fail to mate. We measured tooth number in >700 genotypes derived from a multiparental advanced-intercross population, mapping three moderate-effect loci contributing to trait heritability. Two quantitative trait loci (QTLs) coincide with previously identified intra- and interspecific sex comb QTL, but such overlap can be explained by chance alone, in part because of the broad swathes of the genome implicated by earlier, low-resolution QTL scans. Our mapped QTL regions encompass 70–124 genes, but do not include those genes known to be involved in developmental specification of the comb. Nonetheless, we identified plausible candidates within all QTL intervals, and used RNA interference to validate effects at four loci. Notably, *TweedleS* expression knockdown substantially reduces tooth number. The genes we highlight are strong candidates to harbor segregating, functional variants contributing to sex comb tooth number.

## Introduction

Understanding the evolutionary and developmental basis of novel morphological, physiological and behavioral traits is a critical problem in genetics. Such traits can represent fundamental differences between closely related species, and dissecting their genetic and developmental emergence can help to uncover the evolutionary processes that occur during species divergence. In combination with elucidating the genetic basis of phenotypic differences between species, it is additionally essential to characterize the variation that segregates within natural populations, and articulate the relationship between inter- and intraspecific trait variation. One of the oldest debates in evolutionary biology is over the forces that maintain standing phenotypic variation within species (Provine, 1971; Lewontin, 1974). One set of models posit that variation is largely a result of intermediate-frequency polymorphisms that are actively maintained in populations by balancing selection (Barton and Keightley, 2002; Turelli and Barton, 2004). These models are consistent with Darwin's idea that variation between species is simply an extension of preexisting variation segregating within populations (Darwin, 1859; Lewontin, 1974). An alternative series of models propose that the bulk of variation is due to continuously arising, individually rare deleterious mutations (Johnson and Barton, 2005). If true, species diversification occurs by the rapid fixation of highly infrequent spontaneous advantageous mutations, such that there are qualitative differences between variants leading to species differences and variants that segregate within species. One effective way to determine the evolutionary processes acting on complex trait variation is to experimentally identify and characterize the underlying molecular genetic basis of multiple quantitative trait loci (QTLs), identify the genes involved and ultimately detect the molecular signatures of specific selective forces at functional loci (Mitchell-Olds *et al.*, 2007).

Male secondary sexual traits—courtship displays, body coloration, ornaments, armaments/weapons and so on—provide abundant examples of evolutionary novelty, with many instances of rapidly evolving traits discriminating closely related species (Eberhard, 1985; Andersson, 1994). Evolution of these traits is ultimately because of competition for reproduction, driven by sexual selection on variation in male traits via processes such as female choice, male-to-male competition and sexual conflict (Parker, 1979; Andersson, 1994; Arnqvist, 1998; Hosken and Stockley, 2004; Emlen *et al.*, 2005; Emlen, 2008). By studying the genetic control of male-specific secondary sexual traits within and between species, we can learn about the processes and selective forces leading to diversification.

In *Drosophila* one such male-limited secondary sexual trait is the sex comb, a cluster of specialized bristles present on the forelegs of some species within the *melanogaster* and *obscura* groups (Kopp and True, 2002; Kopp, 2011). Among closely related species within these clades, sex combs vary radically in the number of ‘teeth' on each comb, the morphology of the teeth, the orientation of the comb relative to the leg axis and the position and number of comb arrays on the forelegs (Kopp and True, 2002; Barmina and Kopp, 2007; Kopp, 2011).

The function of the sex comb has been examined in a number of studies that have physically and genetically ablated the entire comb, or experimentally reduced the number of teeth on each comb, to assess the behavior of manipulated males and their ability to copulate. Early studies showed reduced copulation/insemination success in *Drosophila mauritiana*, *D. persimilis*, *D. pseudoobscura* and *D. simulans* males that had forelegs severed to remove the region carrying the sex comb (Spieth, 1952; Coyne, 1985), and in *D. melanogaster* and *D. simulans* males where comb teeth were physically removed with forceps (Cook, 1977). A reduction in the mating success of combless *D. melanogaster* males was also observed by Ng and Kopp (2008) using genetic ablation of the sex comb. Recently, an elegant study employed precise laser ablation of comb teeth in both *D. melanogaster* and *D. bipectinata*, finding that the removal of all comb teeth in either species effectively eliminates copulation in both no choice and competitive trials (Hurtado-Gonzales *et al.*, 2015). The failure of males to copulate in these experiments appears to be because of the inability of the male to grasp the female's abdomen/genitalia as a result of the absence of the sex comb teeth (Hurtado-Gonzales *et al.*, 2015).

If removal of all comb teeth is deleterious to males, the question arises of whether the precise number of teeth that a male possesses is related to function, and is under selection. Surveys of several populations and strains of *D. melanogaster* show that males generally have 7–14 teeth per comb (Ahuja and Singh, 2008; Sharma *et al.*, 2011; Snook *et al.*, 2013). Ahuja and Singh (2008) employed divergent artificial selection and generated high and low sex comb tooth populations. Competing pairs of males with differing tooth numbers (means of 5.45 and 3.31 teeth) from a low-selected strain revealed that those with more teeth were significantly more likely to achieve a successful copulation, suggesting sexual selection against very low sex comb tooth number (Ahuja and Singh, 2008). Nevertheless, an experiment that maintained *D. melanogaster* and *D. pseudoobscura* populations for many generations under regimes allowing various levels of sexual selection failed to lead to any correlated selection for differences in sex comb tooth number (Snook *et al.*, 2013). Studies to assess the level of sexual selection acting on the sex comb in natural, wild-caught populations (by counting teeth on the sex combs of males captured while copulating or not) have shown similarly mixed outcomes. Markow *et al.* (1996) found that *D. simulans* with relatively fewer teeth have greater copulation success. Conversely, Polak *et al.* (2004) found that *D. bipectinata* had greater mating success when the number of teeth on one of the three comb tooth rows was higher. No evidence for an effect of the number of teeth on mating was evident for *D. pseudoobscura* (Markow *et al.*, 1996). Thus, although the comb as a whole appears to be critical for male mating, the relationship between mating success and the precise number of comb teeth is not yet clear.

The sex comb has become a valuable developmental genetics model for the rapid evolution of trait variation because of its exceptional morphological diversity among related species (Kopp, 2011). The diversity of comb morphology throughout the *melanogaster* and *obscura* groups is strongly related to the expression of the HOX transcription factor *Sex combs reduced* (*Scr*). *Scr* is upregulated in the sex comb precursor cells, often showing sexually dimorphic expression in species with sex combs (higher *Scr* expression in males), with no such increased expression or dimorphism in species lacking combs (Barmina and Kopp, 2007). *Scr* serves to activate *doublesex* (*dsx*) in the tarsal region during the late larval stage, and *dsx* expression subsequently initiates sex-specific development of the comb, and defines its morphology (Tanaka *et al.*, 2011). The role of *dsx* in the morphogenesis of the sex comb appears to be at least partly carried out through a repression of *dachshund* (*dac*) as Dac is absent from the sex comb during pupal development (Atallah *et al.*, 2009, 2014,). An unanswered question is whether these high-level developmental regulatory genes that are responsible for development of the sex comb also segregate for allelic variation that confers intraspecific variation in sex comb morphology.

In this study we sought to map loci contributing to variation in sex comb tooth number variation in the model *D. melanogaster* system. Our goal was to ask whether genomic regions contributing to intraspecific trait variation were consistent with those for interspecific variation mapped in prior studies. In addition, we ask whether genes involved in the developmental specification of the comb are also involved in intraspecific variation in comb tooth number. Using genotypes derived from the *Drosophila* Synthetic Population Resource (DSPR) (King *et al.*, 2012b), a large set of recombinant inbred lines (RILs) derived from a multiparental advanced-generation intercross, we were able to map QTLs with greater resolution than in previous genome-wide studies (True *et al.*, 1997; Macdonald and Goldstein, 1999; Nuzhdin and Reiwitch, 2000; Kopp *et al.*, 2003; Tatsuta and Takano-Shimizu, 2006). The three QTLs we map all make small but significant contributions to trait heritability, and our data suggest that these loci may each harbor a series of alleles affecting phenotype. The overlap we see between our QTLs and those mapped in interspecific crosses can be explained by chance alone, and no mapped QTL overlaps with *a priori* developmental candidate genes (for example, *dac*, *dsx*, *Scr*). We functionally test at least one gene under each mapped QTL using tissue-specific RNA interference (RNAi), validating effects at the genes *disco-r*, *poly U binding factor 68kD* (*pUf68*), *scribbled* (*scrib*) and *TweedleS* (*TwdlS*). These genes are strong candidates to harbor functional polymorphisms that causally affect sex comb tooth number variation within *D. melanogaster*, and provide a set of plausible candidate loci for future functional and behavioral testing.

## Materials and methods

### QTL mapping population and phenotyping

We mapped QTLs contributing to sex comb tooth number variation using genotypes derived from the DSPR (FlyRILs.org; King *et al.*, 2012b). The DSPR consists of two sets of RILs (pA and pB), each descended from an advanced-generation intercross of eight founder lines, seven specific to a panel (A1–A7 or B1–B7) and one common to both panels (AB8). Each recombinant population was maintained as a pair of replicate subpopulations (pA1, pA2, pB1, pB2) at large population size for 50 generations to expand the genetic map. RILs were subsequently established from each subpopulation via full-sib mating, and genotyped by restriction site-associated DNA sequencing (Baird *et al.*, 2008). Using full resequencing data for the 15 founder lines and hidden Markov model we assign to each region in each RIL probabilities that the genomic segment is derived from each of the eight founders. For 95% of all positions over all RILs the most likely founder has a probability of >0.95, allowing accurate inference of the mosaic founder haplotype structure of each RIL. King *et al.* (2012b) provide full details of the development and properties of the DSPR RILs.

For the current experiment we generated and assayed the male F_1_ progeny of crosses between independent pairs of pA and pB RILs, in each case crossing pA RIL females to pB RIL males. Given the unidirectional nature of these crosses, and our use of male offspring, we are only able to address X-linked variation segregating in the pA population. The crosses maintained the subpopulation structure of the DSPR by crossing pA1 to pB2 flies (subsequently referred to as ‘subpopulation 1') and pA2 to pB1 flies (‘subpopulation 2'), and we arbitrarily chose to cross RILs within the same subpopulation to avoid using any given RIL in more than one cross (that is, we carried out crosses pA1_1_ x pB2_1_ to pA1_*n*_ × pB2_*n*_, and crosses pA2_1_ × pB1_1_ to pA2_*n*_ × pB1_*n*_). See King *et al.* (2012a) for a comparison of the power and resolution of QTL mapping when phenotyping the F_1_ progeny of RIL-by-RIL crosses *versus* phenotyping RILs directly.

RIL flies were allowed to lay eggs in narrow, polystyrene *Drosophila* vials containing cornmeal–yeast–molasses media. Flies were allowed to lay for up to 2 days, and adults were periodically cleared from vials to maintain roughly equal egg density across experimental vials. After 8–10 days, we harvested 10 virgin female flies from each pA RIL, and 10 male flies from each pB RIL under CO_2_ anesthesia. These flies—the parents of the experimental genotypes—were allowed to recover for at least 1 day before crossing, and flies were again allowed up to 2 days to lay eggs before being removed from vials. After 12–14 days, we collected >10 F_1_ experimental males from each cross vial and stored at –20 °C until phenotyping. One foreleg from each test male was removed (we made no attempt to record whether we scored the left or right leg), mounted in mineral oil on a glass side and tooth number was manually scored at × 40 total magnification.

We counted the number of teeth present on one sex comb for each of 10 males from each experimental cross vial. This modest within-genotype replication is because of our principal interest in estimating the mean phenotype associated with each founder haplotype in the mapping population, rather than in providing highly accurate estimates of genotype means. We scored males from 713 genotypes across 6 experimental batches, collecting data from 57 to 168 genotypes per batch. For the majority of genotypes experimental males were derived from a single experimental batch/vial. However, 56 genotypes were generated and tested as described above in more than one batch, and the correlation between means calculated separately for each batch is high (Pearson's *r*=0.70, *P*<10^−8^, Supplementary File S1), giving us confidence in our phenotypes. Raw phenotype scores are presented in Supplementary File S2.

### Sex comb tooth number heritability

Following the method described in Marriage *et al.* (2014), the broad-sense heritability of the trait was estimated separately for each subpopulation by calculating the genetic and phenotypic variance components from a linear model of the form: *Y*_*ijk*_=*μ*+*b*_*i*_+*g*_*ij*_+*ɛ*_*ijk*_, where *Y*_*ijk*_ is the *k*th observation of the *j*th genotype in the *i*th batch, *μ* is the grand mean, *b*_*i*_ is the random effect of batch, *g*_*ij*_ is the random effect of RIL cross genotype nested within batch and *ɛ*_*ijk*_ is the error term. The components were calculated in R (http://www.R-project.org) using the lme and VarCorr functions in the nlme package (Pinheiro *et al.*, 2011). Note that as individuals of the same genotype were typically raised in the same vial, our heritability estimates will inevitably include some environmental effects. Heritability of the pA × pB cross progeny genotype means used for mapping was estimated as the genetic variance component divided by the total variance of genotype means.

### QTL mapping

**Figure 1:**
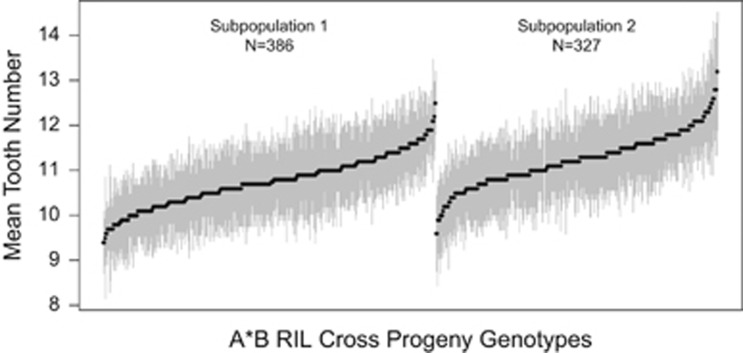
Variation in sex comb tooth number in the DSPR. We generated heterozygous genotypes by intercrossing 713 independent pairs of DSPR RILs, resulting in 386 genotypes from subpopulation 1 (A1 virgin females × B2 males) and 327 from subpopulation 2 (A2 virgin females × B1 males). For each genotype we counted the number of comb teeth on one leg from each of multiple male flies (mean=11 flies/genotype), and present the mean tooth number on a single comb for each genotype (filled circles) and 1 s.d. (vertical lines).

The general approach to mapping QTL in the DSPR is described in detail in King *et al.* (2012a, 2012bss), the full analytical model is provided in King *et al.* (2014) and we implemented mapping routines within the DSPRqtl R package (github.com/egking/DSPRqtl; FlyRILs.org). Briefly, we regress mean male sex comb tooth number for each genotype on the 16 additive probabilities that correspond to the probabilities that maternal RIL is derived from each of the eight pA founders, and the probabilities that paternal RIL is derived from each of the eight pB founders. We additionally include subpopulation as a covariate as there is a difference between the two subpopulations in the average tooth number per genotype: subpopulation 1 mean (±1 s.d.)=10.79±0.565, subpopulation 2 mean (±1 s.d.)=11.19±0.598 (Figure 1). To estimate a genome-wide significance threshold for QTL identification we used 1000 permutations of the data (Churchill and Doerge, 1994). Finally, we used 3-LOD support intervals to approximate 95% confidence intervals on the true positions of causative loci, as for QTLs of modest effects mapped in the pA × pB cross design, typical 2-LOD drops may underestimate the appropriate interval (King *et al.*, 2012a). Raw QTL mapping output is provided in Supplementary File S3.

### Sex comb QTLs mapped in prior studies

A number of previous studies have mapped QTLs contributing to variation in male sex comb tooth number within and between *Drosophila* species. Nuzhdin and Reiwitch (2000) identified two X-linked QTLs segregating within a small panel of 98 *D. melanogaster* RILs derived from a pair of inbred strains. Kopp *et al.* (2003) found eight markers associated with tooth number variation in a set of 144 *D. melanogaster* RILs derived from a single pair of individuals collected from nature. True *et al.* (1997) used a backcross mapping design to identify two QTLs contributing to the divergence between *D. simulans* and *D. mauritiana*, and in a similar study Macdonald and Goldstein (1999) found four QTLs between *D. simulans* and *D. sechellia*. Finally, Tatsuta and Takano-Shimizu (2006) found five QTLs for sex comb tooth number in an F_2_ cross between *D. simulans* strains.

In order to assess any overlap in QTL locations across studies we determined the position of previously mapped sex comb QTLs on our genetic map (see Supplementary File S4). When QTL positions were provided as cytological locations in these studies, we converted to nucleotide positions (*D. melanogaster* reference genome release 5) using the map conversion tools on FlyBase (dos Santos *et al.*, 2015). When the QTL positions were given as the intervals between gene-based markers, we simply used the known positions of these genes from FlyBase. Nucleotide positions were subsequently converted to genetic positions on the DSPR map by virtue of the high-density genotyping conducted on all DSPR RILs, and accurate estimation of genetic distances throughout the genome (King *et al.*, 2012a, b).

We note that unlike the other four studies, Kopp *et al.* (2003) used a marker-based rather than an interval-based mapping methodology and provide only the positions of significant markers. Given the haplotype structure of the mapping population, 95% confidence intervals on QTL locations are likely to be substantially larger than reported.

Difficulties arose in two cases where Tatsuta and Takano-Shimizu (2006) mapped QTLs to intervals overlapping the proximal or distal boundaries of a large paracentric inversion on chromosome 3R that is fixed between *D. melanogaster* and the three species of the *D. simulans* clade (*D. mauritiana*, *D. sechellia* and *D. simulans*). The positions of this inversion were taken from Ranz *et al.* (2007), and for each of the *D. simulans* QTLs in question the equivalent region in *D. melanogaster* is represented by a pair of noncontiguous genomic segments.

### Functional validation of plausible candidate genes

Assessment of the genes within mapped QTL intervals led us to functionally test effects on the sex comb of five genes using the bipartite Gal4-UAS RNAi system. To drive knockdown we used *rn-Gal4* (Bloomington *Drosophila* Stock Center number 8142), an enhancer trap present just upstream of the *rotund* transcription unit, that expresses Gal4 at the position of the presumptive tarsus in the leg disc (St Pierre *et al.*, 2002). In combination with the female splice isoform of the *transformer* (*tra*) gene under the control of the UAS promoter, the *rn-Gal4* driver has been used to genetically ablate the sex comb in *Drosophila* males (Ng and Kopp, 2008). We also employed three *dsx-Gal4* drivers, gifts from the Baker (Ashburn, VA, USA) and Goodwin labs (Oxford, UK). These are targeted knock-ins of Gal4 into the native *doublesex* gene and express Gal4 in a pattern that generally recapitulates that of endogenous *dsx* expression (Rideout *et al.*, 2010; Robinett *et al.*, 2010; Pan *et al.*, 2011). Regulation of *dsx* expression is critical for the proper development of the sex comb (Tanaka *et al.*, 2011).

Transgenic RNAi Project (TRiP) UAS-RNAi and co-isogenic control strains were obtained from the BDSC (Bloomington, IN, USA), specifically stock numbers 36303 (chromosome 3 landing site control strain), 35786 (*UAS-GFP* control), 35788 (*UAS-Luciferase* control), 41683 (*UAS-disco-r-RNAi*), 31960 (*UAS-Dsp1-RNAi*), 25951 and 34785 (*UAS-pUf68-RNAi*), 29552 (*UAS-scrib-RNAi*) and 61864 (*UAS-TwdlS-RNAi*). We also obtained UAS-RNAi stocks, and the appropriate control strains, from the Vienna *Drosophila* Resource Center (Vienna, Austria) (Dietzl *et al.*, 2007); 20144 (*UAS-pUf68-RNAi*), a ‘GD' line harboring a *P*-element transgene, 109796 (*UAS-pUf68-RNAi*) and 105414 (*UAS-scrib-RNAi*), ‘KK' lines harboring phiC31-based UAS transgenes, 60000 (*w*^*1118*^ host strain for GD library) and 60100 (KK landing site control strain). All Gal4 and UAS transgenes are inserted into autosomal locations (see Supplementary File S5 for additional details.)

We generated RNAi knockdown/control male genotypes by crossing 10 virgin females from each UAS/control strain to 10 males from each Gal4 strain, establishing multiple vials per cross over multiple blocks. For each experimental male, the phenotype was scored as the average number of teeth over both sex combs (raw phenotype scores are presented in Supplementary File S6). The final sample size for each genotype varies because of marked differences in the number of males of the desired genotype emerging from each cross vial, likely a consequence of pre-adult lethality for some of the knockdown genotypes generated. Every experimental block included control genotypes to assess any block-to-block variation in phenotype.

### Fly rearing

All stock maintenance and rearing of experimental individuals was carried out at 25 °C and 50% relative humidity with a 12 h light/12 h dark cycle.

### Statistical analysis

All statistics were carried out using core, or custom written, routines in the R statistical programming language (http://www.R-project.org).

## Results

### Heritability

Variation in male sex comb tooth number is substantial in the DSPR (Figure 1), with heterozygous pA × pB cross progeny genotype means ranging from 9.4 to 13.2 teeth per sex comb. This variation is similar to the range of 9.2–12.9 teeth observed across 32 lines of *D. melanogaster* derived from a worldwide set of collection sites (Ahuja and Singh, 2008), and similar to the 7–13 teeth (Sharma *et al.*, 2011) and ~11 teeth (Snook *et al.*, 2013) present in laboratory-adapted outbred populations. On average, genotype means in subpopulation 1 are lower than those in subpopulation 2 (Welch's *t*=−9.1, *P*<10^−15^). Given the subpopulations are derived from the same sets of founders, but were maintained independently for 50 generations before the creation of RILs, differences most likely reflect variation in the founder composition of the subpopulations (see King *et al.*, 2012b).

The broad-sense heritability for sex comb tooth number was estimated as 0.23 and 0.22 for subpopulations 1 and 2, respectively. Variance due to batch was <1% in each case. These heritability values are somewhat higher than, although not inconsistent with, narrow-sense heritability estimates of 0.07–0.21 from the response to artificial selection for increased and decreased comb tooth number in *D. melanogaster* (Ahuja and Singh, 2008). For QTL mapping we employ genotype means, averaging over all 10 sex comb measurements per genotype, and the broad-sense heritability of these mean phenotypes is 0.77 and 0.75 for subpopulations 1 and 2, respectively. The effect of environmental variation on sex comb tooth number has been reported to be much greater than that of genetic variation, with genetic and environmental coefficients of variation of 2.96–3.36 and 7.87–7.99, respectively (Ahuja and Singh, 2008). We see comparable values in this study (genetic coefficient of variation=4.5, environmental coefficients of variation=8.2), and hence by measuring multiple individuals we reduce the effect of environmental noise, and our mean sex comb phenotype has much higher heritability.

### Sex comb QTLs

**Figure 2:**
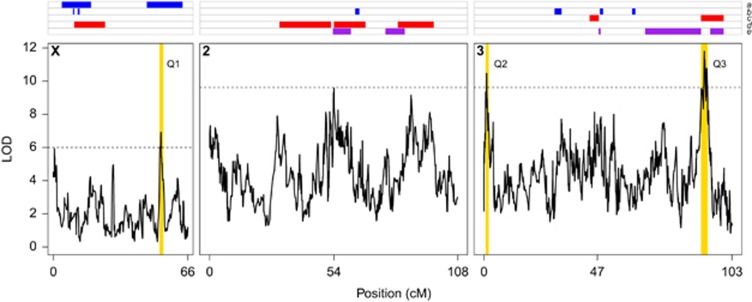
Genome scan for QTLs contributing to sex comb tooth number variation. The bottom three panels show a genome scan in the DSPR for sex comb tooth number QTLs (solid curve) along each of the major fly chromosomes. Genetic positions 54 and 47 on chromosomes 2 and 3, respectively, represent the positions of centromeres. The horizontal dashed lines represent genome-wide 5% permutation thresholds (X: LOD=6.0, autosomes: LOD=9.6). The 3-LOD drop intervals implicated by the three QTLs we map (Q1, Q2, Q3) are highlighted as vertical yellow bars. The top panel shows the positions of sex comb QTLs mapped in five previous studies: *a*, within *D. melanogaster* (blue; Nuzhdin and Reiwitch, 2000); *b*, within *D. melanogaster* (blue; Kopp *et al.*, 2003); *c*, between *D. simulans* and *D. mauritiana* (red; True *et al.*, 1997); *d*, between *D. simulans* and *D. sechellia* (red; Macdonald and Goldstein, 1999); *e*, within *D. simulans* (purple; Tatsuta and Takano-Shimizu, 2006). In placing the QTLs mapped in studies *c–e* on our map we have accounted for the large chromosome *3R* paracentric inversion that is fixed between *D. melanogaster* and *D. mauritiana*/*D. sechellia*/*D. simulans* (see Materials and methods).

Using a similar approach to recent studies using the DSPR (Kislukhin *et al.*, 2013; King *et al.*, 2014) we mapped QTL contributing to variation in sex comb tooth number across 713 pA × pB cross progeny male genotypes. Three QTLs survived a permutation-derived 5% genome-wide threshold (Figure 2 and Table 1); Q1 is X-linked, and Q2 and Q3 are near the opposite ends of chromosome 3 on 3L and 3R, respectively. QTLs explain 4.4–7.4% of the genetic variation for sex comb tooth number and—assuming additivity—collectively explain 18.4% of the total genetic variation for the trait (Table 1). Given the fairly large sample size employed, we do not expect substantial upward bias in QTL effect estimates—the Beavis effect (Beavis, 1994; Xu, 2003)—nonetheless, the values we estimate should be considered as upper bounds on the variance explained by these loci.

**Figure 3:**
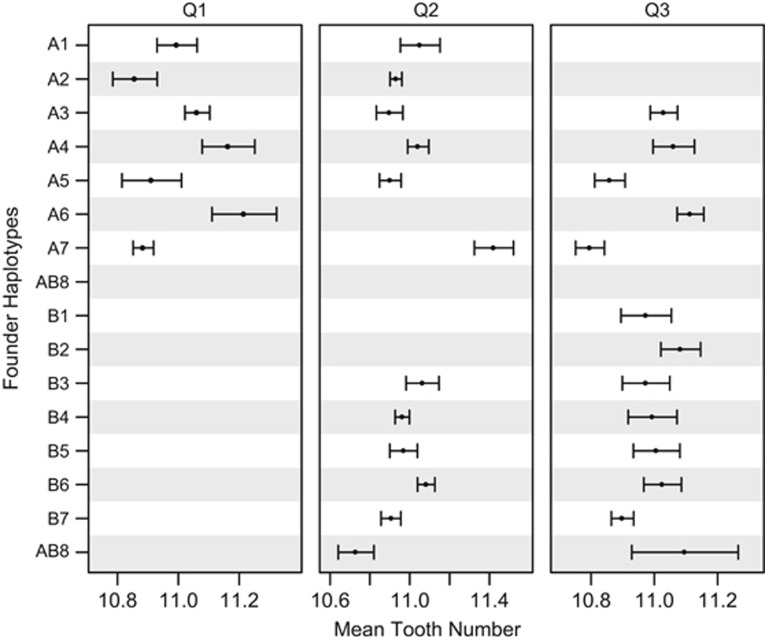
Founder haplotype effects for mapped QTLs. Phenotype means (and s.e.) are presented for each founder at the peak position of each QTL. Data are only presented for founders present in at least 10 RILs at a probability of >0.95. AB8 was used to found both synthetic populations, and hence is depicted twice in the figure. Given that we phenotyped males resulting from crosses between population A females and population B males, for the X-linked Q1 only means for population A founders can be estimated.

Standard QTL mapping studies attempt to identify causative loci that segregate between a pair of parental genotypes (see Mackay, 2001). Thus, only a tiny fraction of the allelic variation in a population is interrogated, and such studies cannot evaluate the extent to which causative loci harbor a series of functional alleles with different effects on phenotype. With the DSPR we have the opportunity to evaluate the phenotypic effects of up to 15 alleles. Figure 3 shows the estimated founder haplotype effects at each of the mapped QTLs, and does not suggest a clear grouping of the founders into two groups, the pattern expected under simple biallelism. Although the patterns we observe could be because of allelic heterogeneity at single causative genes under each QTL, given the size of the implicated intervals (0.61–0.99 megabases; Table 1), it is not unlikely that multiple linked genes contribute to the effects estimated at each QTL. In addition, the variation in founder genotype frequency in the DSPR, where the frequency of each founder deviates from the expected 1/8 throughout the genome (King *et al.*, 2012b), could make effects difficult to estimate and complicate their interpretation.

### Overlap of sex comb QTLs among studies

Two previous studies have mapped QTL for sex comb tooth number in small panels of *D. melanogaster* RILs (Nuzhdin and Reiwitch, 2000; Kopp *et al.*, 2003). The positions of these QTLs are depicted in the top panel of Figure 2 (blue bars, rows *a* and *b*). One of the two X-linked QTLs mapped in Nuzhdin and Reiwitch (2000) (Figure 2, row *a*) overlaps with Q1 mapped here, but otherwise none of the QTLs previously observed to contribute to sex comb tooth number variation within *D. melanogaster* overlap those we map in the DSPR. As 9.2% of the physical genome harbors previously mapped intraspecific QTL, finding one 1- Mb DSPR QTL that overlaps such a region is not unexpected by chance (Poisson probability, *k*=1, λ=0.092, *P*=0.09). We see the same result when considering that previously mapped intraspecific QTLs encompass 12.9% of the genetic map length (*P*=0.11). True differences in the architecture of trait variation among the three *D. melanogaster* mapping populations could obviously explain this lack of overlap, and clearly studies initiated from 2 to 4 alleles (Nuzhdin and Reiwitch, 2000; Kopp *et al.*, 2003) may not have captured functional alleles present in the DSPR. Differences in power among the studies could also plausibly explain the differences we see. In this context, previous simulations of the power of the DSPR pA × pB cross design to identify QTLs contributing 5–10% of the variation is 68–95% (King *et al.*, 2012a). Thus, if the genetic architecture of sex comb tooth number is dominated by small- to modest-effect QTLs, insufficient power may lead to studies routinely identifying different subsets of QTLs.

Two studies have previously mapped between-species variation for sex comb tooth number (Figure 2, top panel, red bars, rows *c* and *d*), using *D. simulans–D. sechellia* recombinants (Macdonald and Goldstein, 1999), and *D. simulans–D. mauritiana* recombinants (True *et al.*, 1997). The largest-effect QTL mapped by True *et al.* (1997), positioned over the chromosome 3 centromere (Figure 2), has been further resolved by Graze *et al.* (2007) who implicated a small handful of genes as plausibly contributing to the difference in sex comb tooth number between *D. simulans* and *D. mauritiana*. Only our Q3 overlaps an interspecific sex comb QTL, mapping at the same location as the small-effect QTL identified by True *et al.* (1997) (Figure 2). The apparent differences in the architecture of within- and between-species variation we observe could be real or could be due to the technical differences in QTL mapping methods described above.

Collectively, across five previous studies, 53.3% (49.5%) of the physical (genetic) map has been implicated in the genetic control of sex comb tooth number variation, and by random chance we would expect that two of the QTLs we map would overlap these regions (Poisson probability, *k*=2, λ=0.533/0.495, *P*=0.08/0.07), and this is what we observe. Interestingly, no previous studies have mapped a factor influencing sex comb tooth number variation to the left end of chromosome 3L where we identify QTL Q2 (Figure 2).

### Candidate sex comb tooth number genes

Several genes are known to affect the specification and development of the sex comb, such as *Scr* (Lewis *et al.*, 1980a, b; Barmina and Kopp, 2007; Tanaka *et al.*, 2011), *dac* (Atallah *et al.*, 2014), the sex determination pathway genes *dsx* and *transformer 2*, *tra2* (Baker and Ridge, 1980; Tanaka *et al.*, 2011) and a series of genes named based on their effect on the sex comb (*Additional sex combs*, *extra sex combs*, *multi sex combs*, *Posterior sex combs*, *Sex comb on midleg*, *Sex combs extra* and *super sex combs*). Although some of these genes, such as *dsx* and *Scr* (Graze *et al.*, 2007), have been implicated within QTL intervals identified in previous mapping studies, none of them are present within the three QTLs we map in the DSPR (Supplementary File S7).

In the absence of classic candidate genes within the modest number of genes in our QTL intervals (Table 1), we employed two strategies to attempt to define plausible candidates for subsequent testing. First, to identify any genes in FlyBase with a putative or functionally characterized role in sex comb development, we used the controlled vocabulary search terms ‘sex comb', ‘sex comb tooth' and ‘sex comb development' to extract a total of 93 genes tagged with one or more of these terms (see Supplementary File S8). The genes *Dsp1* (*Dorsal switch protein 1*) and *pUf68* (*poly U binding factor 68kD*) were previously annotated as having some role in sex comb formation (Decoville *et al.*, 2001; Quinn *et al.*, 2004), and are present within QTL intervals Q1 and Q2, respectively. Second, we exploited data from a microarray study (Barmina *et al.*, 2005) that identified two independent sets of genes of interest (see Supplementary Tables 1 and 3 from Barmina *et al.*, 2005): (1) 16 genes differentially expressed between male, sex comb-bearing T1 pupal legs (forelegs) and male, non-sex comb-bearing T2 pupal legs and (2) 143 genes differentially expressed between male T1 pupal legs and female, non-sex comb-bearing T1 pupal legs. *TwdlS* (*TweedleS*) was differentially expressed between male T1 and T2 legs, and is under Q3. Genes *disco-r* (*disco-related*) and *scrib* (*scribbled*) were differentially expressed between male T1 and female T1 legs, and are present within the Q1 and Q3 intervals, respectively.

We attempted to functionally validate the effect of these five genes on the number of sex comb tooth in *D. melanogaster* using the bipartite Gal4-UAS RNAi system. The two types of Gal4 drivers used—*rn-Gal4* and *dsx-Gal4—*are expected to knock down expression of target genes in slightly different spatiotemporal patterns, but in both cases include the presumptive sex comb precursor region of the foreleg.

**Figure 4:**
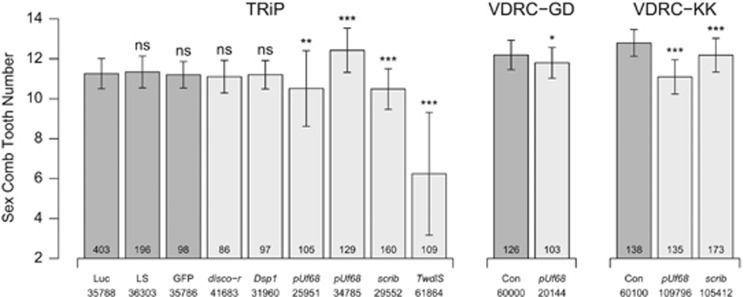
*rn-Gal4* RNAi knockdown of plausible candidate genes implicated by QTL. Each bar represents the mean tooth number on a single comb (±1 s.d.) for several *rn-Gal4/UAS* male RNAi knockdown (light gray bars) and control genotypes (dark gray bars). The name of each UAS control or gene is provided beneath each bar along with the stock number of the UAS strain in the Bloomington *Drosophila* Stock Center (TRiP) or the Vienna *Drosophila* Resource Center (VDRC). The sample size per genotype is presented at the bottom of each bar. The phenotype of each knockdown was compared with the appropriate control via Welch's *t*-tests (NS, not significant, *P<0.01, **P<0.0005, ***P<10^–10^). For TRiP lines we tested three control strains—‘Luc' (*UAS-Luciferase*), ‘LS' (landing site for phiC31-based transgenesis) and ‘GFP' (*UAS-GFP*)—and here present statistical comparison of each gene knockdown to ‘Luc'. The ‘Luc' control genotype was tested in all experimental blocks, and no significant block-to-block variation was observed for this genotype. For VDRC-GD and -KK lines the control genotype is listed as ‘Con'.

**Figure 5:**
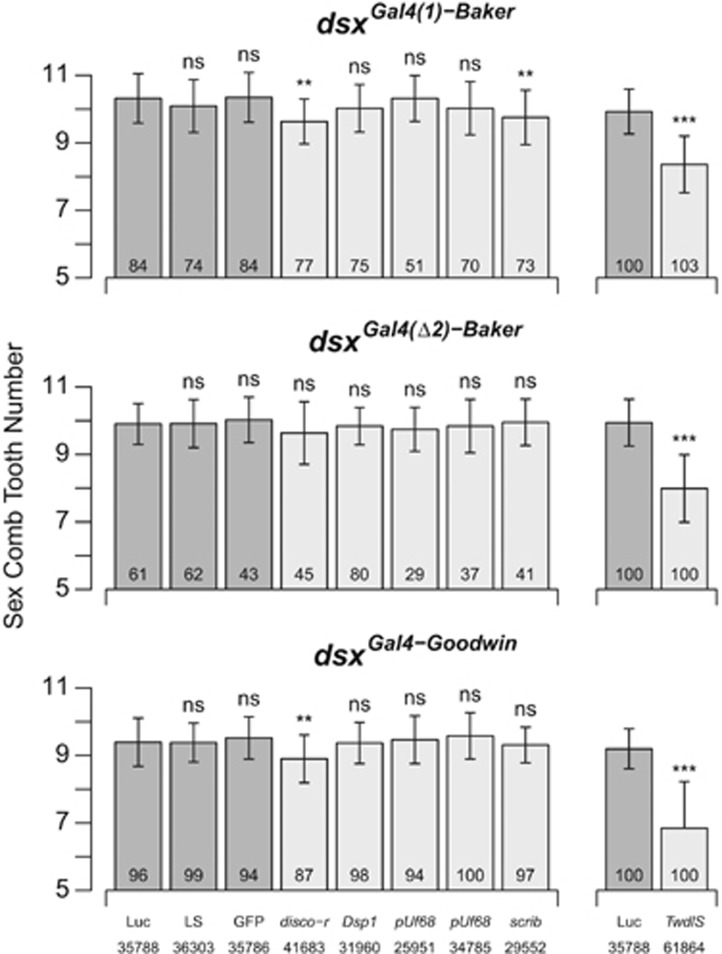
*dsx-Gal4* RNAi knockdown of plausible candidate genes implicated by QTLs. We used three different *dsx-Gal4* lines to knock down candidate gene expression; *dsx*^*Gal4(1)*^ is described in Robinett *et al.* (2010), *dsx*^*Gal4(*Δ*2)*^ in Pan *et al.* (2011) and *dsx*^*Gal4*^ in Rideout *et al.* (2010). The eight genotypes on the left of each panel were generated and scored in one experimental block, whereas the two genotypes on the right were generated/scored in an independent block. Because of a slight but significant block-to-block difference in the mean phenotype of the control genotype containing *UAS-Luciferase* (‘Luc'), knockdown genotypes were compared only with controls generated and scored contemporaneously. See Figure 4 for details of the labels and presentation of the data. NS, not significant, **P<0.0005, ***P<10^–10^.

Knockdown of *disco-r* (under Q1) shows no effect with the *rn-Gal4* driver (Figure 4), but we see a reduction in the number of sex comb teeth with two of the three *dsx-Gal4* drivers (Figure 5). Knockdown of *Dsp1* (also under Q1) shows no effect on the phenotype with any driver (Figures 4 and 5), potentially implying it has no role in the sex comb, although we cannot discount the possibility that *Dsp1* expression is simply not being reduced because of technical factors (see Booker *et al.*, 2011).

For *pUf68* (under Q2) we see a significant change in phenotype for all *rn-Gal4* knockdowns tested (Figure 4), but none of the *dsx-Gal4* knockdowns (Figure 5). The situation is complicated by the observation that although three of the *pUf68 rn-Gal4* knockdowns yield lower sex comb tooth numbers than the control, in one case (construct ID 34785) we see significantly more teeth (Figure 4). We regenerated this latter *pUf68* RNAi genotype using the reciprocal cross (crossing males from the UAS strain to females from the Gal4 strain) that serves to swap the X chromosome present in the test males, but observed the same increase in comb tooth number relative to the control (Supplementary File S9). Given the irregular formation of the comb in *pUf68* RNAi animals, and the variable size and melanization of the teeth (Supplementary File S10), some of the variations across genotypes may simply represent experimental difficulty counting the number of teeth present.

For *scrib* (under Q3) there is a significant reduction in sex comb tooth number with *rn-Gal4* (Figure 4), and we also observe a reduction with one of the three *dsx-Gal4* drivers (Figure 5). For *TwdlS* (also under Q3) we see a dramatic reduction in sex comb tooth number across all drivers (Figures 4 and 5 and Supplementary File S10).

## Discussion

Darwin posited the conversion of variation within species into adaptive differences among species. If genes that contribute to polymorphism within a species are generally also those that influence a trait difference between species, there should be significant overlap between mapped intra- and interspecific QTL intervals. Several previous studies have observed QTL overlap in both within and between species (Nuzhdin and Reiwitch, 2000; Fishman *et al.*, 2002; Lexer *et al.*, 2005; Hall *et al.*, 2006; Wittkopp *et al.*, 2009; McNeil *et al.*, 2011; Groot *et al.*, 2013), supporting the idea that interspecific differences are not fundamentally distinct from variation within species in many instances. However, at least one study failed to identify any overlap in the positions of QTLs mapped within and between species (Gleason and Ritchie, 2004). QTL overlap does not provide information on whether those alleles responsible for species differentiation were present as standing variation in the ancestral populations from which the species arose, or were generated by new mutations. However, it can suggest that extant allelic variation within a species, and differences between species, reside at the same loci, demonstrating shared genetic control of trait variation within and between species.

Here, we genetically tested Darwin's idea using variation in the number of teeth on the sex comb, a male-specific sexual structure in *Drosophila*. The gross morphology of the sex comb has rapidly diverged among closely related species (Kopp, 2011), there is variation among the four *melanogaster* complex species in the number of comb teeth (True *et al.*, 1997) and there is some evidence that tooth number is under selection (see, for example, Ahuja and Singh, 2008). Using a multiparental, advanced intercross mapping population, and phenotyping well over 7000 animals, we mapped three QTLs contributing to sex comb tooth variation within *D. melanogaster*. We subsequently examined overlap between the positions of QTLs contributing to sex comb tooth number in the DSPR, and the positions of intra- and interspecific sex comb QTLs identified in previous work using the *melanogaster* group of Drosophilid species (True *et al.*, 1997; Macdonald and Goldstein, 1999; Nuzhdin and Reiwitch, 2000; Kopp *et al.*, 2003; Tatsuta and Takano-Shimizu, 2006). The level of overlap between the QTLs that we identified and those identified in five previous mapping studies is not strong, and indeed is consistent with chance. Nonetheless, neither is overlap absent. Q1 overlaps with one of the two X-linked QTLs mapped by Nuzhdin and Reiwitch (2000) in a cross between *D. melanogaster* strains (Figure 2), and Q3 overlaps with a QTL mapped to the end of chromosome 3R by True *et al.* (1997) in a between-species mapping panel (Figure 2). It is possible we have replicated effects at the same genes in these instances, but available data preclude any confident test of this assertion. The low resolution of early QTL mapping studies, which ensures large fractions of the genome are implicated in the control of trait variation, and the imperfect power to map small-effect QTLs in the DSPR, which means only a subset of the functional polymorphisms are resolved, makes assessing the similarity of intra- and interspecific genetic architecture for sex comb tooth number technically challenging.

One biological limitation of the within- and between-species comparison we present is the lack of an interspecific cross involving *D. melanogaster*. Such crosses are not possible because of hybrid inviability and sterility. A more appropriate test may be to build on the work of Tatsuta and Takano-Shimizu (2006) and generate high-resolution mapping data within *D. simulans*, allowing direct comparison with interspecific variation between *D. simulans* and either *D. mauritiana* or *D. sechellia*. Recent advances in high-throughput genotyping (see, for example, Andolfatto *et al.*, 2011) allow very large mapping populations in non-model systems to be genetically analyzed (Huang and Erezyilmaz, 2015). This offers the possibility of mapping sex comb number QTL in non-*D. melanogaster* recombinants with sufficient resolution that the similarity of trait architecture within and between species can be compared with confidence.

In contrast to previous mapping studies, we were able to use the DSPR to map modest-effect QTL to short, statistically defined regions, providing the opportunity to uncover likely candidate genes. None of the three QTLs we map implicate *dac*, *dsx*, *Scr* or *tra2* (Supplementary File S7), the four genes that have been shown to be involved in the specification and development of the sex comb (Baker and Ridge, 1980; Lewis *et al.*, 1980a, b; Barmina and Kopp, 2007; Tanaka *et al.*, 2011; Atallah *et al.*, 2014). Although not formally significant at a 5% permutation-derived threshold (Churchill and Doerge, 1994), we observed three additional peaks in the LOD (logarithm (base 10) of odds) score profile that are close to genome-wide significance (X, 0.5 cM; 2L, 54.0 cM; and 2R, 87.5 cM; Figure 2 and Supplementary File S3). These peaks could represent true, small-effect QTLs or simply be spurious. Regardless, 3-LOD support intervals about these peaks also do not implicate *dac*, *dsx*, *Scr* or *tra2*. These results imply that key players in sex comb developmental patterning either do not contribute to natural variation in sex comb tooth number in the DSPR or were undetectable in our study by virtue of having relatively small individual effects or by segregating for very rare functional alleles not captured in the DSPR.

Despite the absence of *a priori* candidate genes beneath mapped QTL intervals, we were able to leverage other data to define one or two plausible candidates for each QTL, and used RNAi directed to the region of the foreleg from which the sex comb is derived to show that four of these genes affect sex comb tooth number. *disco-r* is present within the interval implicated by X-linked QTL Q1, was shown to have lower expression in male compared with female pupal forelegs (Barmina *et al.*, 2005) and has been suggested to be involved in leg development (Grubbs *et al.*, 2013). *pUf68* is implicated by QTL Q2 on chromosome 3. This gene is differentially expressed between adult virgin males and females (McIntyre *et al.*, 2006), and a loss-of-function mutation leads to malformed sex combs (Quinn *et al.*, 2004). The product of *pUf68* is known to regulate alternative splicing (Van Buskirk and Schupbach, 2002), and is required for splicing of the M1 intron from the *tra2* primary transcript, although it does not appear to contribute to Tra2 control of *dsx* splicing (Wang *et al.*, 2013). *scrib* is present within QTL Q3 on chromosome 3 and, similar to *disco-r*, has been shown to have lower gene expression in male forelegs compared with female forelegs (Barmina *et al.*, 2005). This gene is a tumor suppressor involved in the control of epithelial cell proliferation (Bilder *et al.*, 2000) and epithelial cell polarity (Bilder and Perrimon, 2000), and has been shown to regulate asymmetric cell division in *Drosophila* neuroblasts (Albertson and Doe, 2003). In addition, *scrib* was isolated during a *P*-element mutagenesis screen to identify genes showing defects in sensory bristle number (Norga *et al.*, 2003). Finally, *TwdlS* is also present under Q3, and was previously shown to be differentially expressed between male T1 and T2 legs (Barmina *et al.*, 2005). Little is known about the functional role of this gene, although based on analyses of other genes in the same family, the family is insect specific and may have a role in assembly of the cuticle (Guan *et al.*, 2006). In our study the effect on sex comb tooth number of a knockdown of *TwdlS* expression was pronounced (Figures 4 and 5) and the magnitude of the phenotypic change was greater than for any other gene we tested. In addition, for all *dsx-Gal4-*driven *TwdlS* knockdowns, we noted that the mutant male flies appeared to completely lack proper external genitalia, suggesting the gene may play a critical, higher-order role in sexual trait determination in *D. melanogaster*. One caveat to these RNAi-based functional tests is the possibility for off-target, nonspecific effects of the RNAi reagents to lead to false positives (Ma *et al.*, 2006). However, such effects should be minimized in cases where multiple transgenes yield similar results.

In summary, in this study we have used a combination of high-resolution QTL mapping, published expression data and RNAi-based functional tests to implicate several short regions of the *Drosophila* genome, and a handful of loci, in the genetic control of male sex comb tooth number variation. Three of the candidate sex comb genes that we identify—*disco-r*, *scrib* and *TwdlS—*have not previously been shown to affect the morphology of the sex comb, and given their reported expression differences between sexes or segments in the *Drosophila* foreleg (Barmina *et al.*, 2005), may be expected to segregate for functional regulatory polymorphisms that affect sex comb tooth number. Identification of the precise causative variants may be accomplished by association mapping in natural populations of flies (see, for example, Bastide *et al.*, 2013), although the large environmental component to the variation of sex comb tooth number, the subtle effects of the QTLs we map and the possibility that QTLs are multiallelic imply that the kinds of sample size typically employed in the human disease association mapping literature will be required (that is, tens of thousands of individuals). Alternatively, it is now becoming feasible in *D. melanogaster* to employ CRISPR-Cas9 genome editing to compare different, putatively functional alleles in an otherwise standardized background and directly test the effects of specific genomic intervals, genes and nucleotide positions.

## Data archiving

All phenotype data collected as part of this study are available as Supplementary Information files. All data on the DSPR project is available at FlyRILs.org.

## Figures and Tables

**Table 1:** Details of QTLs mapped for sex comb tooth number variation in the DSPR

*Name*	*Chr*	*LOD score*	*Peak cM (3-LOD CI)*^a^	*Peak Mb (3-LOD CI)*^a^	*Number of genes*^b^	*Percent of H*^*2*^^c^
Q1	X	6.9	53.0 (52.3–54.2)	16.05 (15.81–16.42)	70	4.4
Q2	3L	10.5	1.1 (0.6–1.9)	1.48 (1.22–1.90)	124	6.6
Q3	3R	11.8	91.5 (90.1–92.9)	22.57 (22.09–23.08)	114	7.4

Abbreviations: CI, confidence interval; Chr, chromosome; DSPR, *Drosophila* Synthetic Population Resource; LOD, logarithm (base 10) of odds; QTL, quantitative trait locus.

a 3-LOD CI indicates the 3-LOD support interval of the QTLs. Physical positions are given based on release 5 of the *Drosophila* reference genome.

b The number of protein-coding genes in the 3-LOD support interval.

c The percentage of broad-sense heritability of genotype means explained by the QTLs.

## References

[bib1] Ahuja A, Singh RS. (2008). Variation and evolution of male sex combs in Drosophila: nature of selection response and theories of genetic variation for sexual traits. Genetics 179: 503–509. 18493067 10.1534/genetics.107.086363 PMC2390627

[bib2] Albertson R, Doe CQ. (2003). Dlg, Scrib and Lgl regulate neuroblast cell size and mitotic spindle asymmetry. Nat Cell Biol 5: 166–170. 12545176 10.1038/ncb922

[bib3] Andersson M. (1994) Sexual Selection. Princeton University Press: Princeton, NJ.

[bib4] Andolfatto P, Davison D, Erezyilmaz D, Hu TT, Mast J, Sunayama-Morita T et al. (2011). Multiplexed shotgun genotyping for rapid and efficient genetic mapping. Genome Res 21: 610–617. 21233398 10.1101/gr.115402.110 PMC3065708

[bib5] Arnqvist G. (1998). Comparative evidence for the evolution of genitalia by sexual selection. Nature 393: 784–786.

[bib6] Atallah J, Liu NH, Dennis P, Hon A, Larsen EW. (2009). Developmental constraints and convergent evolution in Drosophila sex comb formation. Evol Dev 11: 205–218. 19245551 10.1111/j.1525-142X.2009.00320.x

[bib7] Atallah J, Vurens G, Mavong S, Mutti A, Hoang D, Kopp A. (2014). Sex-specific repression of dachshund is required for Drosophila sex comb development. Dev Biol 386: 440–447. 24361261 10.1016/j.ydbio.2013.12.017

[bib8] Baird NA, Etter PD, Atwood TS, Currey MC, Shiver AL, Lewis ZA et al. (2008). Rapid SNP discovery and genetic mapping using sequenced RAD markers. PLoS One 3: e3376. 18852878 10.1371/journal.pone.0003376 PMC2557064

[bib9] Baker BS, Ridge KA. (1980). Sex and the single cell. I. On the action of major loci affecting sex determination in Drosophila melanogaster. Genetics 94: 383–423. 6771185 10.1093/genetics/94.2.383 PMC1214149

[bib10] Barmina O, Gonzalo M, McIntyre LM, Kopp A. (2005). Sex- and segment-specific modulation of gene expression profiles in Drosophila. Dev Biol 288: 528–544. 16269142 10.1016/j.ydbio.2005.09.052

[bib11] Barmina O, Kopp A. (2007). Sex-specific expression of a HOX gene associated with rapid morphological evolution. Dev Biol 311: 277–286. 17868668 10.1016/j.ydbio.2007.07.030

[bib12] Barton NH, Keightley PD. (2002). Understanding quantitative genetic variation. Nat Rev Genet 3: 11–21. 11823787 10.1038/nrg700

[bib13] Bastide H, Betancourt A, Nolte V, Tobler R, Stobe P, Futschik A et al. (2013). A genome-wide, fine-scale map of natural pigmentation variation in Drosophila melanogaster. PLoS Genet 9: e1003534. 23754958 10.1371/journal.pgen.1003534 PMC3674992

[bib14] Beavis WD. (1994). The power and deceit of QTL experiments: lessons from comparative QTL studies. Proceedings of the 49th Annual Corn and Sorghum Industry Research Conference. American Seed Trade Association: Washington, DC. pp250–266.

[bib15] Bilder D, Li M, Perrimon N. (2000). Cooperative regulation of cell polarity and growth by Drosophila tumor suppressors. Science 289: 113–116. 10884224 10.1126/science.289.5476.113

[bib16] Bilder D, Perrimon N. (2000). Localization of apical epithelial determinants by the basolateral PDZ protein Scribble. Nature 403: 676–680. 10688207 10.1038/35001108

[bib17] Booker M, Samsonova AA, Kwon Y, Flockhart I, Mohr SE, Perrimon N. (2011). False negative rates in Drosophila cell-based RNAi screens: a case study. BMC Genomics 12: 50. 21251254 10.1186/1471-2164-12-50 PMC3036618

[bib18] Churchill GA, Doerge RW. (1994). Empirical threshold values for quantitative trait mapping. Genetics 138: 963–971. 7851788 10.1093/genetics/138.3.963 PMC1206241

[bib19] Cook RM. (1977). Behavioral role of the sexcombs in Drosophila melanogaster and Drosophila simulans. Behav Genet 7: 349–357. 411471 10.1007/BF01077448

[bib20] Coyne JA. (1985). Genetic studies of three sibling species of Drosophila with relationship to theories of speciation. Genet Res 46: 169–192. 3936752 10.1017/s0016672300022643

[bib21] Darwin C. (1859) The Origin of Species. John Murray: London, UK.

[bib22] Decoville M, Giacomello E, Leng M, Locker D. (2001). DSP1, an HMG-like protein, is involved in the regulation of homeotic genes. Genetics 157: 237–244. 11139505 10.1093/genetics/157.1.237 PMC1461500

[bib23] Dietzl G, Chen D, Schnorrer F, Su KC, Barinova Y, Fellner M et al. (2007). A genome-wide transgenic RNAi library for conditional gene inactivation in Drosophila. Nature 448: 151–156. 17625558 10.1038/nature05954

[bib24] dos Santos G, Schroeder AJ, Goodman JL, Strelets VB, Crosby MA, Thurmond J et al. (2015). FlyBase: introduction of the Drosophila melanogaster Release 6 reference genome assembly and large-scale migration of genome annotations. Nucleic Acids Res 43: D690–D697. 25398896 10.1093/nar/gku1099 PMC4383921

[bib25] Eberhard WG. (1985) Sexual Selection and Animal Genitalia. Harvard University Press: Cambridge, MA.

[bib26] Emlen DJ. (2008). The evolution of animal weapons. Annu Rev Ecol Evol Syst 39: 387–413.

[bib27] Emlen DJ, Marangelo J, Ball B, Cunningham CW. (2005). Diversity in the weapons of sexual selection: horn evolution in the beetle genus Onthophagus (Coleoptera: Scarabaeidae). Evolution 59: 1060–1084. 16136805

[bib28] Fishman L, Kelly AJ, Willis JH. (2002). Minor quantitative trait loci underlie floral traits associated with mating system divergence in Mimulus. Evolution 56: 2138–2155. 12487345 10.1111/j.0014-3820.2002.tb00139.x

[bib29] Gleason JM, Ritchie MG. (2004). Do quantitative trait loci (QTL) for a courtship song difference between Drosophila simulans and D. sechellia coincide with candidate genes and intraspecific QTL? Genetics 166: 1303–1311. 15082549 10.1534/genetics.166.3.1303 PMC1470780

[bib30] Graze RM, Barmina O, Tufts D, Naderi E, Harmon KL, Persianinova M et al. (2007). New candidate genes for sex-comb divergence between Drosophila mauritiana and Drosophila simulans. Genetics 176: 2561–2576. 17565959 10.1534/genetics.106.067686 PMC1950655

[bib31] Groot AT, Staudacher H, Barthel A, Inglis O, Schofl G, Santangelo RG et al. (2013). One quantitative trait locus for intra- and interspecific variation in a sex pheromone. Mol Ecol 22: 1065–1080. 23294019 10.1111/mec.12171

[bib32] Grubbs N, Leach M, Su X, Petrisko T, Rosario JB, Mahaffey JW. (2013). New components of Drosophila leg development identified through genome wide association studies. PLoS One 8: e60261. 23560084 10.1371/journal.pone.0060261 PMC3613359

[bib33] Guan X, Middlebrooks BW, Alexander S, Wasserman SA. (2006). Mutation of TweedleD, a member of an unconventional cuticle protein family, alters body shape in Drosophila. Proc Natl Acad Sci USA 103: 16794–16799. 17075064 10.1073/pnas.0607616103 PMC1636534

[bib34] Hall MC, Basten CJ, Willis JH. (2006). Pleiotropic quantitative trait loci contribute to population divergence in traits associated with life-history variation in Mimulus guttatus. Genetics 172: 1829–1844. 16361232 10.1534/genetics.105.051227 PMC1456280

[bib35] Hosken DJ, Stockley P. (2004). Sexual selection and genital evolution. Trends Ecol Evol 19: 87–93. 16701234 10.1016/j.tree.2003.11.012

[bib36] Huang Y, Erezyilmaz D. (2015). The genetics of resistance to Morinda fruit toxin during the postembryonic stages in Drosophila sechellia. G3 (Bethesda) 5: 1973–1981. 26224784 10.1534/g3.114.015073 PMC4592979

[bib37] Hurtado-Gonzales JL, Gallaher W, Warner A, Polak M. (2015). Microscale laser surgery demonstrates the grasping function of the male sex combs in Drosophila melanogaster and Drosophila bipectinata. Ethology 121: 45–56.

[bib38] Johnson T, Barton N. (2005). Theoretical models of selection and mutation on quantitative traits. Philos Trans R Soc Lond B Biol Sci 360: 1411–1425. 16048784 10.1098/rstb.2005.1667 PMC1569515

[bib39] King EG, Kislukhin G, Walters KN, Long AD. (2014). Using Drosophila melanogaster to identify chemotherapy toxicity genes. Genetics 198: 31–43. 25236447 10.1534/genetics.114.161968 PMC4174942

[bib40] King EG, Macdonald SJ, Long AD. (2012a). Properties and power of the Drosophila Synthetic Population Resource for the routine dissection of complex traits. Genetics 191: 935–949. 22505626 10.1534/genetics.112.138537 PMC3389985

[bib41] King EG, Merkes CM, McNeil CL, Hoofer SR, Sen S, Broman KW et al. (2012b). Genetic dissection of a model complex trait using the Drosophila Synthetic Population Resource. Genome Res 22: 1558–1566. 22496517 10.1101/gr.134031.111 PMC3409269

[bib42] Kislukhin G, King EG, Walters KN, Macdonald SJ, Long AD. (2013). The genetic architecture of methotrexate toxicity is similar in Drosophila melanogaster and humans. G3 (Bethesda) 3: 1301–1310. 23733889 10.1534/g3.113.006619 PMC3737169

[bib43] Kopp A. (2011). Drosophila sex combs as a model of evolutionary innovations. Evol Dev 13: 504–522. 23016935 10.1111/j.1525-142X.2011.00507.x PMC3462374

[bib44] Kopp A, Graze RM, Xu SZ, Carroll SB, Nuzhdin SV. (2003). Quantitative trait loci responsible for variation in sexually dimorphic traits in Drosophila melanogaster. Genetics 163: 771–787. 12618413 10.1093/genetics/163.2.771 PMC1462463

[bib45] Kopp A, True JR. (2002). Evolution of male sexual characters in the oriental Drosophila melanogaster species group. Evol Dev 4: 278–291. 12168620 10.1046/j.1525-142x.2002.02017.x

[bib46] Lewis RA, Kaufman TC, Denell RE, Tallerico P. (1980a). Genetic analysis of the Antennapedia gene complex (Ant-C) and adjacent chromosomal regions of Drosophila melanogaster. I. Polytene chromosome segments 84b-D. Genetics 95: 367–381. 17249041 10.1093/genetics/95.2.367 PMC1214232

[bib47] Lewis RA, Wakimoto BT, Denell RE, Kaufman TC. (1980b). Genetic analysis of the Antennapedia gene complex (Ant-C) and adjacent chromosomal regions of Drosophila melanogaster. II. Polytene chromosome segments 84A-84B1,2. Genetics 95: 383–397. 17249042 10.1093/genetics/95.2.383 PMC1214233

[bib48] Lewontin RC. (1974) The Genetic Basis of Evolutionary Change. Columbia University Press: New York, NY.

[bib49] Lexer C, Rosenthal DM, Raymond O, Donovan LA, Rieseberg LH. (2005). Genetics of species differences in the wild annual sunflowers, Helianthus annuus and H. petiolaris. Genetics 169: 2225–2239. 15545657 10.1534/genetics.104.031195 PMC1449618

[bib50] Ma Y, Creanga A, Lum L, Beachy PA. (2006). Prevalence of off-target effects in Drosophila RNA interference screens. Nature 443: 359–363. 16964239 10.1038/nature05179

[bib51] Macdonald SJ, Goldstein DB. (1999). A quantitative genetic analysis of male sexual traits distinguishing the sibling species Drosophila simulans and D. sechellia. Genetics 153: 1683–1699. 10581276 10.1093/genetics/153.4.1683 PMC1460840

[bib52] Mackay TF. (2001). Quantitative trait loci in Drosophila. Nat Rev Genet 2: 11–20. 11253063 10.1038/35047544

[bib53] Markow T, Bustoz D, Pitnick S. (1996). Sexual selection and a secondary sexual character in two Drosophila species. Animal Behav 52: 759–766.

[bib54] Marriage TN, King EG, Long AD, Macdonald SJ. (2014). Fine-mapping nicotine resistance loci in Drosophila using a multiparent advanced generation inter-cross population. Genetics 198: 45–57. 25236448 10.1534/genetics.114.162107 PMC4174953

[bib55] McIntyre LM, Bono LM, Genissel A, Westerman R, Junk D, Telonis-Scott M et al. (2006). Sex-specific expression of alternative transcripts in Drosophila. Genome Biol 7: R79. 16934145 10.1186/gb-2006-7-8-r79 PMC1779584

[bib56] McNeil CL, Bain CL, Macdonald SJ. (2011). Multiple quantitative trait loci influence the shape of a male-specific genital structure in Drosophila melanogaster. G3 (Bethesda) 1: 343–351. 22384345 10.1534/g3.111.000661 PMC3276151

[bib57] Mitchell-Olds T, Willis JH, Goldstein DB. (2007). Which evolutionary processes influence natural genetic variation for phenotypic traits? Nat Rev Genet 8: 845–856. 17943192 10.1038/nrg2207

[bib58] Ng CS, Kopp A. (2008). Sex combs are important for male mating success in Drosophila melanogaster. Behav Genet 38: 195–201. 18213513 10.1007/s10519-008-9190-7

[bib59] Norga KK, Gurganus MC, Dilda CL, Yamamoto A, Lyman RF, Patel PH et al. (2003). Quantitative analysis of bristle number in Drosophila mutants identifies genes involved in neural development. Curr Biol 13: 1388–1396. 12932322 10.1016/s0960-9822(03)00546-3

[bib60] Nuzhdin SV, Reiwitch SG. (2000). Are the same genes responsible for intra- and interspecific variability for sex comb tooth number in Drosophila? Heredity 84: 97–102. 10692016 10.1038/sj.hdy.6886400

[bib61] Pan Y, Robinett CC, Baker BS. (2011). Turning males on: activation of male courtship behavior in Drosophila melanogaster. PLoS One 6: e21144. 21731661 10.1371/journal.pone.0021144 PMC3120818

[bib62] Parker GA. (1979) Sexual selection and sexual conflict In:Blum MS, Blum NA(eds) Sexual Selection and Reproductive Competition in Insects. Academic Press: New York, NY. pp 123–166.

[bib63] Pinheiro J, Bates D, DebRoy S, Sarkar D, Team RDC. (2011). nlme: linear and nonlinear mixed effects models. R package version 31-101.

[bib64] Polak M, Starmer WT, Wolf LL. (2004). Sexual selection for size and symmetry in a diversifying secondary sexual character in Drosophila bipectinata duda (Diptera: Drosophilidae). Evolution 58: 597–607. 15119443

[bib65] Provine WB. (1971) The Origins of Theoretical Population Genetics. The University of Chicago Press: Chicago, IL.

[bib66] Quinn LM, Dickins RA, Coombe M, Hime GR, Bowtell DD, Richardson H. (2004). Drosophila Hfp negatively regulates dmyc and stg to inhibit cell proliferation. Development 131: 1411–1423. 14993190 10.1242/dev.01019

[bib67] Ranz JM, Maurin D, Chan YS, von Grotthuss M, Hillier LW, Roote J et al. (2007). Principles of genome evolution in the Drosophila melanogaster species group. PLoS Biol 5: e152. 17550304 10.1371/journal.pbio.0050152 PMC1885836

[bib68] Rideout EJ, Dornan AJ, Neville MC, Eadie S, Goodwin SF. (2010). Control of sexual differentiation and behavior by the doublesex gene in Drosophila melanogaster. Nat Neurosci 13: 458–466. 20305646 10.1038/nn.2515 PMC3092424

[bib69] Robinett CC, Vaughan AG, Knapp JM, Baker BS. (2010). Sex and the single cell. II. There is a time and place for sex. PLoS Biol 8: e1000365. 20454565 10.1371/journal.pbio.1000365 PMC2864297

[bib70] Sharma MD, Tregenza T, Hosken DJ. (2011). Sex combs, allometry, and asymmetry in Drosophila. Biol J Linn Soc 103: 923–934.

[bib71] Snook RR, Gidaszewski NA, Chapman T, Simmons LW. (2013). Sexual selection and the evolution of secondary sexual traits: sex comb evolution in Drosophila. J Evol Biol 26: 912–918. 23496332 10.1111/jeb.12105

[bib72] Spieth HT. (1952). Mating behavior within the genus Drosophila (Diptera). Bull Am Mus Nat Hist 99: 395–474.

[bib73] St Pierre SE, Galindo MI, Couso JP, Thor S. (2002). Control of Drosophila imaginal disc development by rotund and roughened eye: differentially expressed transcripts of the same gene encoding functionally distinct zinc finger proteins. Development 129: 1273–1281. 11874922 10.1242/dev.129.5.1273

[bib74] Tanaka K, Barmina O, Sanders LE, Arbeitman MN, Kopp A. (2011). Evolution of sex-specific traits through changes in HOX-dependent doublesex expression. PLoS Biol 9: e1001131. 21886483 10.1371/journal.pbio.1001131 PMC3160335

[bib75] Tatsuta H, Takano-Shimizu T. (2006). Genetic architecture of variation in sex-comb tooth number in Drosophila simulans. Genet Res 87: 93–107. 16709273 10.1017/S0016672306008111

[bib76] True JR, Liu JJ, Stam LF, Zeng ZB, Laurie CC. (1997). Quantitative genetic analysis of divergence in male secondary sexual traits between Drosophila simulans and Drosophila mauritiana. Evolution 51: 816–832. 10.1111/j.1558-5646.1997.tb03664.x 28568599

[bib77] Turelli M, Barton NH. (2004). Polygenic variation maintained by balancing selection: pleiotropy, sex-dependent allelic effects and G x E interactions. Genetics 166: 1053–1079. 15020487 10.1093/genetics/166.2.1053 PMC1470722

[bib78] Van Buskirk C, Schupbach T. (2002). Half pint regulates alternative splice site selection in Drosophila. Dev Cell 2: 343–353. 11879639 10.1016/s1534-5807(02)00128-4

[bib79] Wang S, Wagner EJ, Mattox W. (2013). Half pint/Puf68 is required for negative regulation of splicing by the SR splicing factor Transformer2. RNA Biol 10: 1396–1406. 23880637 10.4161/rna.25645 PMC3817160

[bib80] Wittkopp PJ, Stewart EE, Arnold LL, Neidert AH, Haerum BK, Thompson EM et al. (2009). Intraspecific polymorphism to interspecific divergence: genetics of pigmentation in Drosophila. Science 326: 540–544. 19900891 10.1126/science.1176980

[bib81] Xu S. (2003). Theoretical basis of the Beavis effect. Genetics 165: 2259–2268. 14704201 10.1093/genetics/165.4.2259 PMC1462909

