# Supplementary material for: Genetic dissection of intraspecific variation in a male-specific sexual trait in Drosophila melanogaster
Source: Heredity (Edinb). 2016 Aug 17;117(6):417–26. doi: 10.1038/hdy.2016.63 (PMC5117841; doi:10.1038/hdy.2016.63)

**Supplementary File S1** Correlation of genotype means measured over experimental batches. Fifty-six DSPR pA by pB cross genotypes were generated and assayed (tooth number measured for a single comb from 10 males) over more than one experimental batch. The correlation among means calculated independently for each batch is high (Pearson's *r* = 0.70, *p* < 10^–8^).

**Supplementary File S2** Raw sex comb tooth number phenotype data from DSPR. The "Subpop" column gives the subpopulation each cross belongs to (subpopulation 1 = pA1 by pB2 crosses; subpopulation 2 = pA2 by pB1 crosses). The "Batch" column describes the experimental batch (1-6) from which the data is derived. The "matRIL" and "patRIL" columns define the stock numbers of the maternal and paternal, respectively, DSPR RILs from which the experimental cross genotypes are derived. The "RepMaleNum" column assigns a number (1-10) to each male assayed per genotype per block. The "NumTeeth" column is the number of teeth on a single sex comb from the assayed male. (Available for separate download as a *.txt file.)

**Supplementary File S3** LOD scores for genomewide QTL scan in the DSPR. The "Chromosome" column indicates the chromosome arm of the position under test (X, 2L, 2R, 3L, and 3R). The "PhysicalPosition" and "GeneticPosition" columns indicate the physical and genetic positions of the site under test, respectively. The physical position is based on release 5 of the *D. melanogaster* reference genome. The "LODscore" column gives the LOD score at each position throughout the genome. (Available for separate download as a *.txt file.)

**Supplementary File S4** Positions of sex comb tooth number QTL mapped in five previous studies. Positions of QTL were derived from the original publications, either as cytological locations, or as the names of gene-based markers flanking the QTL. Information was transposed to physical positions in the *D. melanogaster* genome (Release 5 coordinates) using FlyBase, either by converting cytological positions to nucleotide positions, or by finding the nucleotide positions of the genes in which markers were developed. Finally, nucleotide positions were projected to genetic positions (in cM) in the DSPR. Positions of QTL are depicted in Figure 2.

True et al. (1997; Evolution 51(3), 816-832)

Mapped QTL in an interspecific cross between *D. simulans* and *D. mauritiana*.

QTL1 Flanking gene-based markers = *tra* to *Antp*

Interval (bp) = 3L:16583159 – 3R:2824950

Interval (cM) = 3:43.90 – 47.69

QTL2 Flanking gene-based markers = *Ald* to *janA*

Interval (bp) = 3R:22079791 – 3R:25865255

Interval (cM) = 3:90.11 – 99.56

Macdonald and Goldstein (1999; PMID: 10581276)

Mapped QTL in an interspecific cross between *D. sechellia* and *D. simulans*.

QTL1 Flanking cytological locations = 4F1-F2 to 8B5-B8

Interval (bp) = X:5001140 – X:8827682

Interval (cM) = X:10.26 – 25.46

QTL2 Flanking cytological locations = 29A5-B4 to 36C

Interval (bp) = 2L:8254276 – 2L:17552485

Interval (cM) = 2:30.29 – 52.69

QTL3 Flanking cytological locations = 38E1-E9 to 49F9-F13

Interval (bp) = 2L:20687001 – 2R:9163391

Interval (cM) = 2:53.97 – 67.67

QTL4 Flanking cytological locations = 54B1-B2 to 58A4-B1

Interval (bp) = 2R:13115253 – 2R:17865563

Interval (cM) = 2:81.74 – 97.38

**Supplementary File S4** (continued)

Nuzhdin and Reiwitch (2000; PMID: 10692016)

Mapped QTL in an intraspecific panel of 98 RILs derived from a cross between two *D. melanogaster* strains.

QTL1 Flanking cytological locations = 3E to 6E

Interval (bp) = X:3508246 – X:6927931

Interval (cM) = X:4.20 – 18.45

QTL2 Flanking cytological locations = 12E to 19A

Interval (bp) = X:13975021 – X:19962790

Interval (cM) = X:46.10 – 63.69

Tatsuta and Takano-Shimizu (2006; PMID: 16709273)

Mapped QTL in a number of intraspecific crosses between strains of *D. simulans*. We include data only for those QTL mapped via Composite Interval Mapping in the whole-genome F_2_ cross.

Note that QTL3 and QTL4 overlap the proximal and distal boundaries, respectively, of the large paracentric inversion on chromosome *3R* that discriminates *D. melanogaster* and *D. simulans*. Each QTL implicates a genomic region flanking the inversion, and a region within the inversion, and in *D. melanogaster* these regions are not adjacent to each other. We used the position of gene-based markers flanking the QTL provided, and the estimated positions to the inversion breakpoints to identify the two QTL subregions implicated by each of QTL3 and QTL4.

The breakpoints of this inversion, *In(3R)84F1;93F6-7*, lie between genes *CG2708* and *CG7918*, and between *CG31176* and *CG34034* (Ranz et al., 2007), and we define the proximal boundary as position 3R:3874066 and the distal boundary as position 3R:17554725.

QTL1 Flanking gene-based markers = *Ddc* to *eve*

Interval (bp) = 2L:19116483 – 2R:5868300

Interval (cM) = 2:53.51 – 61.31

QTL2 Flanking gene-based markers = *sli* to *Pcl*

Interval (bp) = 2R:11757000 – 2R:14027495

Interval (cM) = 2:76.33 – 84.70

QTL3 Flanking gene-based markers = *Antp* to *ninaE*

Interval (bp) = 3R:2721975 – 3R:3874066

= 3R:15711977 – 3R:17554725

Interval (cM) = 3:47.65 – 48.13

= 3:66.95 – 72.51

QTL4 Flanking gene-based markers = *hb* to *Ald*

Interval (bp) = 3R:3874066 – 3R:4523544

= 3R:17554725 – 3R:22087313

Interval (cM) = 3:48.13 – 48.44

= 3:72.51 – 90.14

QTL5 Flanking gene-based markers = *Mlc1* to *janA*

Interval (bp) = 3R:23482800 – 3R:25865255

Interval (cM) = 3:93.99 – 99.56**Supplementary File S4** (continued)

Kopp et al. (2003; PMID: 12618413)

Mapped QTL in an intraspecific panel of 144 RILs derived from two, wild-caught *D. melanogaster* individuals.

Note that the positions of QTL in this paper are given as the positions of significant cytological markers. True 95% confidence intervals on QTL locations are likely considerably larger than implied by marker positions.

QTL1 Cytological location = 4E

Interval (bp) = X:4858073 – X:5001139

Interval (cM) = X:9.62 – 10.26

QTL2 Cytological location = 5A

Interval (bp) = X:5369850 – X:5591185

Interval (cM) = X:11.94 – 12.93

QTL3 Cytological location = 47E

Interval (bp) = 2R:7108460 – 2R:7174523

Interval (cM) = 2:63.71 – 63.85

QTL4 Cytological location = 48A

Interval (bp) = 2R:7415288 – 2R:7519380

Interval (cM) = 2:64.31 – 64.51

QTL5 Cytological location = 67C

Interval (bp) = 3L:9540370 – 3L:9858410

Interval (cM) = 3:29.33 – 30.59

QTL6 Cytological location = 67D

Interval (bp) = 3L:9858411 – 3L:10309495

Interval (cM) = 3:30.59 – 32.21

QTL7 Cytological location = 85D

Interval (bp) = 3R:5028243 – 3R:5426095

Interval (cM) = 3:48.71 – 48.95

QTL8 Cytological location = 90EF

Interval (bp) = 3R:13830721 – 3R:13943989

Interval (cM) = 3:62.04 – 62.31

**Supplementary File S5** Details of strains used for RNAi.

Gal4 Strains

*rn-Gal4*

BDSC: 8142

URL: http://flystocks.bio.indiana.edu/Reports/8142.html

Genotype: P{GawB.DeltaS}rn[GAL4-DeltaS]/TM3, P{w[+mC]=ActGFP}JMR2, Ser[1]

Insertion: Chromosome 3

*dsx^Gal4(1)^*

PMID: 20454565

Genotype: w; dsx[Gal4]/TM6B

Insertion: Chromosome 3

*dsx^Gal4(^*^∆^*^2)^*

PMID: 21731661

Genotype: dsx[Gal4(∆2)]/TM6B

Insertion: Chromosome 3

*dsx^Gal4^*

PMID: 20305646

Genotype: dsx[Gal4]

Insertion: Chromosome 3

TRiP UAS Strains

Landing site control strain

BDSC: 36303

URL: http://flystocks.bio.indiana.edu/Reports/36303.html

Genotype: y[1] v[1]; P{y[+t7.7]=CaryP}attP2

Insertion: Chromosome 3

*UAS-GFP*

BDSC: 35786

URL: http://flystocks.bio.indiana.edu/Reports/35786.html

Genotype: y[1] v[1]; P{y[+t7.7] v[+t1.8]=UAS-GFP.VALIUM10}attP2

Insertion: Chromosome 3

*UAS-Luciferase*

BDSC: 35788

URL: http://flystocks.bio.indiana.edu/Reports/35788.html

Genotype: y[1] v[1]; P{y[+t7.7] v[+t1.8]=UAS-LUC.VALIUM10}attP2

Insertion: Chromosome 3

*UAS-disco-r-RNAi*

BDSC: 41683

URL: http://flystocks.bio.indiana.edu/Reports/41683.html

Genotype: y[1] sc[*] v[1]; P{y[+t7.7] v[+t1.8]=TRiP.HMS02247}attP2

Insertion: Chromosome 3

*UAS-Dsp1-RNAi*

BDSC: 31960

URL: http://flystocks.bio.indiana.edu/Reports/31960.html

Genotype: y[1] v[1]; P{y[+t7.7] v[+t1.8]=TRiP.JF02732}attP2

Insertion: Chromosome 3

**Supplementary File S5** (continued)

*UAS-pUf68-RNAi*

BDSC: 25951

URL: http://flystocks.bio.indiana.edu/Reports/25951.html

Genotype: y[1] v[1]; P{y[+t7.7] v[+t1.8]=TRiP.JF01971}attP2

Insertion: Chromosome 3

BDSC: 34785

URL: http://flystocks.bio.indiana.edu/Reports/34785.html

Genotype: y[1] sc[*] v[1]; P{y[+t7.7] v[+t1.8]=TRiP.HMS00094}attP2

Insertion: Chromosome 3

*UAS-scrib-RNAi*

BDSC: 29552

URL: http://flystocks.bio.indiana.edu/Reports/29552.html

Genotype: y[1] v[1]; P{y[+t7.7] v[+t1.8]=TRiP.JF03229}attP2

Insertion: Chromosome 3

*UAS-TwdlS-RNAi*

BDSC: 61864

URL: http://flystocks.bio.indiana.edu/Reports/61864.html

Genotype: y[1] v[1]; P{y[+t7.7] v[+t1.8]=TRiP.HMJ23353}attP40

Insertion: Chromosome 2

VDRC UAS Strains

GD library control strain

VDRC: 60000

URL: http://stockcenter.vdrc.at/control/product/~VIEW_INDEX=0/~VIEW_SIZE=100/~product_id=60000

Genotype: w[1118]

Insertion: NA

KK library landing site control strain

VDRC: 60100

URL: http://stockcenter.vdrc.at/control/product/~VIEW_INDEX=0/~VIEW_SIZE=100/~product_id=60100

Genotype: y w[1118]; P{attP,y[+],w[3']}VIE-260B

Insertion: Chromosome 2

*UAS-pUf68-RNAi*

VDRC: 20144 (GD)

URL: http://stockcenter.vdrc.at/control/product/~VIEW_INDEX=0/~VIEW_SIZE=100/~product_id=20144

Genotype: w[1118]; UAS-pUf68

Insertion: Chromosome 2

VDRC: 109796 (KK)

URL: http://stockcenter.vdrc.at/control/product/~VIEW_INDEX=0/~VIEW_SIZE=100/~product_id=109796

Genotype: y w[1118]; UAS-pUf68

Insertion: Chromosome 2

*UAS-scrib-RNAi*

VDRC: 105414 (KK)

URL: http://stockcenter.vdrc.at/control/product/~VIEW_INDEX=0/~VIEW_SIZE=100/~product_id=105414

Genotype: y w[1118]; UAS-scrib

Insertion: Chromosome 2

**Supplementary File S6** Raw sex comb tooth number phenotype data from Gal4-UAS-RNAi experiments. The "MaleParent" and "FemaleParent" columns give the genotypes of the strains used to create the experimental progeny (see Materials and Methods for details regarding these strains.) The "ExperimentalBlock" column gives the experimental block from which the data is derived. The "RepVial" column gives the replicate cross vial from which experimental males were derived ("NA" values in this column represent instances in which multiple vials were used to collect flies, but records were not maintained regarding which vial each individual was taken from.) The "Individual" column provides a unique code for each individual within each replicate vial and/or genotype. The columns "NumTeeth.Side1", and "NumTeeth.Side2" hold the number of teeth on each sex comb from the assayed male, while "NumTeeth.Average" provides the average for each male. (Available for separate download as a *.txt file.)

**Supplementary File S7** Protein-coding genes implicated by the three sex comb tooth number QTL mapped in the DSPR. The "QTL" column defines which QTL interval a given gene is present within. The "Cytology" and "Nucelotide" columns provide the cytological and nucleotide (Release 5) position of each gene. The "GeneName" column provides the name of the gene. (Available for separate download as a *.txt file.)

**Supplementary File S8** Genes in FlyBase tagged as being involved in sex comb specification. The "CV_Search_Term" column defines the controlled vocabulary search term(s) that tag the gene in FlyBase (sc = "sex comb", scd = "sex comb development", sct = "sex comb tooth"). The "FBID", "CG", "GeneName", and "Symbol" columns are the FlyBase gene ID number, the CG annotation number, the name of the gene, and the gene symbol, respectively. The "NucelotidePosition" column provides the Release 5 position of the each gene.

**Supplementary File S9**  Reciprocal direction Gal4-UAS RNAi experiment for *pUf68*. One of the four *pUf68* RNAi knockdowns with *rn-Gal4* increases sex comb tooth number (construct ID 34785), while the remaining three reduce tooth number. To determine whether this aberrant result was due to the direction of the cross, we generated the same knockdown genotype but used the reciprocal cross (i.e., crossed *rn-Gal4* females to *UAS-pUf68* [34785] males, rather than the reverse as was carried out for all other RNAi experiments). As presented in the plot below, regardless of the cross direction, this *pUf68* RNAi construct leads to an increase in sex comb tooth number. See Figure 4 for details of the labels and presentation of the data.

**Supplementary File S10** Representative images of sex combs from a subset of RNAi genotypes examined in this study.


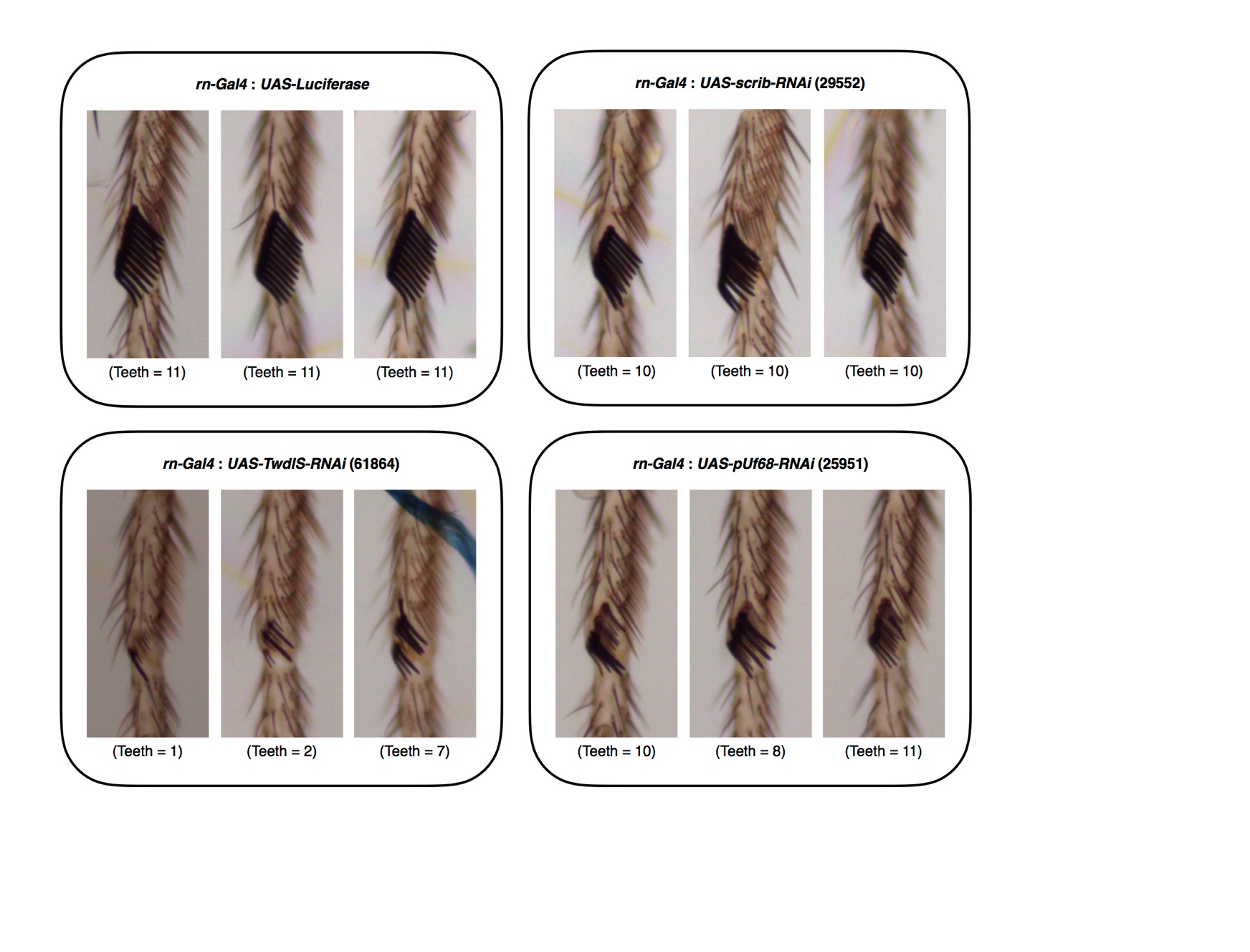

Supplement: Supplementary Information [file hdy201663x1.docx]
